# Midstream Players Determine Population-Level Behavior Change: Social Marketing Research to Increase Demand for Lead-Free Components in Pitcher Pumps in Madagascar

**DOI:** 10.3390/ijerph18147297

**Published:** 2021-07-08

**Authors:** Mahmooda Khaliq, Silvia Sommariva, Adaline M. Buerck, Rinah Rakotondrazaka, Lova Rakotoarisoa, Luke John Paul Barrett, James R. Mihelcic

**Affiliations:** 1 College of Public Health, University of South Florida, 13201 Bruce B Downs Blvd, Tampa, FL 33620, USA; sommarivas@usf.edu; 2 Department of Civil and Environmental Engineering, College of Engineering, University of South Florida, 4202 E Fowler Avenue, ENG 030, Tampa, FL 33620, USA; abuerck@usf.edu (A.M.B.); jm41@usf.edu (J.R.M.); 3 ONG Ranontsika, 22 Bis Rue du Commerce, Ampasimazava, Toamasina 501, Madagascar; rinah@ranontsika.com (R.R.); lova@ranontsika.com (L.R.); ljpb@ranontsika.com (L.J.P.B.)

**Keywords:** social marketing, behavior change, social change, communication, lead exposure, Sub-Saharan Africa, groundwater

## Abstract

Lead (Pb) exposure through water contamination is an important issue at the intersection of public health and water, sanitation, and hygiene (WASH). Behavior-change programs designed to address this pressing problem rarely take a behavioral-science-informed approach, nor do they consider the role of intermediate players who often influence and support behavior change. Social marketing segments the population and focuses on the consumer/user throughout program development and implementation. To illustrate the social marketing process, this cross-sectional, qualitative design study investigates the use of Pb in the construction and maintenance of household pitcher pumps for potable water in Madagascar. A sample of 18 technicians were interviewed on their current practices, motivators, barriers, and communication channels for knowledge exchange. The results reveal the importance of peers, those considered experts or “market mavens”, and the need for information on the dangers of Pb as an outdated practice for any future intervention. This study advances the notion of a design shift within engineering WASH projects, whereby social/behavioral approaches are used to consider the needs, concerns, and current behaviors of the consumer. We also advocate for engaging intermediate players who often influence behavior change in the rollout of an engineering innovation.

## 1. Introduction

Lead (Pb) exposure through water contamination is a key issue at the intersection of public health and water, sanitation, and hygiene (WASH) [1]. A recent report found that around one in three children globally have blood lead levels (BLL) above 5 micrograms per deciliter (µg/dL) [2], a level considered to be a prompt for action, though there is no safe level of Pb for the body; even low BLLs (~1–2 µg/dL) can affect children’s cognitive and social development [3]. The same report identifies pipe replacements as one key long-term solution to eliminating exposure from water sources and suggests that behavior-change interventions should target both the general population and occupationally exposed workers to limit exposure [2]. The present study extends these lines of inquiry to the development of a midstream behavioral intervention to address the problem of Pb in drinking water in the port city of Toamasina, Madagascar. Rather than focus on the end-user, whose health is ultimately compromised by Pb exposure, the midstream approach focuses on behavior change among intermediate players (e.g., pump technicians in Toamasina) who are responsible for the manufacture, installation, and repair of manually operated suction pumps for potable water (i.e., pitcher pumps) in the community.

In Toamasina, access to publicly provided potable water is often unavailable, expensive, and unreliable [4,5,6]. Accordingly, many households use a decentralized, household water supply (e.g., self-supply) and utilize locally produced, manually operated pitcher pumps for their water needs [4,5,6]. There are an estimated 9000 such pitcher pumps in Toamasina serving ~60% of the 280,000 persons in the area [6]. The two locally produced pump valves found in these pumps (described in detail in Mihelcic et al., 2009 [7]) are typically comprised of Pb derived from used lead-acid batteries (ULAB), resulting in Pb leaching into the pumped water [5,6]. Furthermore, this problem is continuous as valves are replaced frequently due to ongoing wear and tear [5]. A study observed that 51% of field measurements of Pb concentrations in this community were above the World Health Organization (WHO) provisional guideline of 10 µg/L in drinking water [5,8]. A recent study modeling the BLLs of children in Toamasina estimates that approximately 34% of Malagasy children in Toamasina may be at risk for elevated BLLs [9].

Prior attempts to solve the issue of Pb in water in Toamasina occurred in 2018. These attempts focused on increasing awareness and educating the general public (households participating in the intervention) and the pump technicians on adverse health effects of Pb. This work found that both audiences were skeptical of a “top-down” approach to encourage behavior change, due to a lack of trust in those conveying the messages and ongoing political elections, which creates instability and presents as a barrier to behavior change. To this end, the study identified a need for a new way to address the problem using a community-based approach, considering the experiences of the main decision-maker, the pump technician, in intervention design. As such, social marketing was identified as the behavior change framework to use in exploring and addressing this gap.

Social marketing adopts commercial marketing tools to change behavior for a positive social impact [10]. It relies, like behavioral economics, on the assumption that information, education, and communication tactics are not enough to change individuals’ behavior and that complexities of human experiences (and barriers) need to be understood and placed at the center of program development in order to achieve long-lasting change [11]. Unlike behavioral economics and the use of nudges, social marketing is a more holistic approach, using tools that go beyond nudges and including promotional tactics, price, and product to encourage behavior change [12]. It has been applied to a variety of fields, including health, environmental protection, and safety/injury prevention [13,14,15,16]. Researchers in this field believe that users will engage in behavior change (e.g., the purchase of a product, use of a service, adoption of a new lifestyle) when they perceive that the benefits are greater than the barriers/challenges faced in making the change [17]. While behavioral economists and the use of nudges focus on changing the social and physical environment, social marketing has traditionally focused on changing behavior with a micro-level approach by focusing narrowly on the end-user. However, recently there has been an increasing push towards “midstream social marketing” [18,19], aiming to promote behavior change by changing the immediate social environment wherein the end-user operates [20,21]. The focus of this study on pump technicians is one step towards this direction.

In WASH, social marketing has been applied to promote conservation of resources, energy, and water through campaigns such as WaterSense by the Environmental Protection Agency (EPA) Partnership Program, an initiative focused on making water savings easy for consumers [14]. A review by Evans et al. (2014) analyzed how social marketing has been used within the context of water and sanitation products, particularly in relation to handwashing, point-of-use water treatment, safe water systems, and latrine adoption. The authors found consistent improvement on behavioral mediators and, in many cases, positive behavior change [22]. Most of the studies identified had focused on promoting change at the household level, while only a few focused on midstream segments. For example, an intervention by CARE, an international global health organization, in southern Kenya focused their efforts on nurses undergoing training as the target audience for a program to improve handwashing [23]. To the best of our knowledge, no study has applied social marketing to promote behavior change among midstream actors (i.e., service personnel such as pump technicians) who can influence and support individual-level behavior change. Considered enactors, pump technicians are directly involved in and influence the behavior-change process [24,25].

The objective of this exploratory study was to apply social marketing principles to understand the mechanisms of behavior change for a midstream actor (pump technicians), and how these are translated into a strategy to increase demand for an engineering innovation. The pump technicians are the key gatekeepers who make the decision to use Pb components, resulting in lead exposure through water for the general population. Therefore, we investigate the barriers and facilitators of switching to lead-free components for this audience, and attempt to understand the mix of communication techniques coupled with community-based activities that encourage the creation of lead-free pumps in Toamasina, Madagascar. This work is especially important because of the complexities of managing lead release from economic activities, whether from large lead smelters [26] or eliminating lead exposure in the unregulated market context often associated with products manufactured, sold, and serviced in low- and middle-income countries (LMICs) by the informal sector. This informal sector consists of small and unorganized individuals who produce ordinary goods and services and operate at the fringes of the formal economy [27,28]. In many LMICs, these small businesses account for over half of total nonagricultural employment [29] and in Sub-Saharan Africa up to 60% of a country’s GDP and as much as 93% of new employment [27]. Thus, these small businesses or midstream actors have vast potential for impactful behavior change for which this study offers a template.

## 2. Materials and Methods

**Figure 1:**
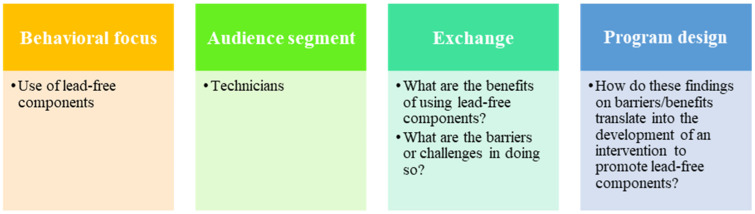
Study framework consisted of four key information factors based in social marketing concepts as applied to this project.

**Figure 2:**
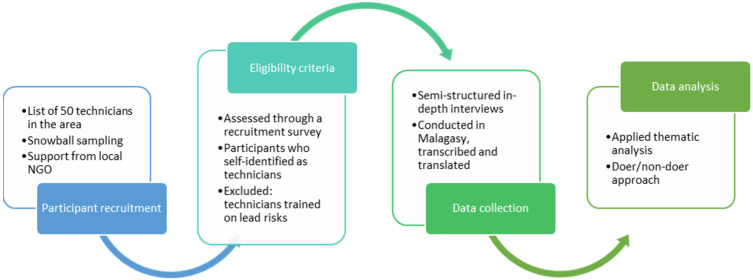
Key steps and information in the study design and methods used for data collection and analysis.

This study utilized a cross-sectional qualitative design to investigate use of Pb in the installation and repair of household pitcher pumps using a social marketing approach (Figure 1). Figure 2 summarizes the study design and methods. The study was deemed to be non-human subjects research (study activities involve analysis of de-identified data) and received approval by the Institutional Review Board at the University of South Florida (IRB Pro# 00041506).

### 2.1. Social Marketing Framework

At the core of social marketing is the idea that individuals will implement something new (this being the adoption of a new behavior or abandoning a habit) if they perceive that the benefits gained are higher than the barriers they must overcome. This concept, also called “consumer exchange”, is both context- and user-specific [30,31,32,33]. For this reason, a key pillar of social marketing is the need to segment and understand the audience. The social marketing approach has implications for program development and implementation: an intervention is not likely to be successful if it is not designed with the user in mind (i.e., it provides clear benefits to its audience). In this respect, it is essential to dig deeper into why a certain behavior is not being adopted by conducting primary data collection with the population of interest. In this study, the behavior of interest, or behavioral focus, was the use of lead-free components for the installation and repair of pitcher pumps. With the pump technicians as the priority population, the intervention design focuses on midstream actors rather than the end-consumer [24]. The goal was to inform the development of an intervention to encourage behavior change by leveraging the perceived benefits of abandoning the use of Pb, or by decreasing the challenges of doing so.

### 2.2. Study Population

Study participants were technicians who install or repair pitcher pumps in Toamasina, Madagascar. Participants self-identified as currently working as technicians and no additional information or proof of their profession was required. Participants who reported having received formal training on the risks associated with Pb in water from the University of South Florida (USF) team as part of a prior project [34] were excluded. Participants who stated that they used Pb components and those who reported not using Pb in their work were included to allow for a doer/non-doer analysis of the issue [35,36].

### 2.3. Sampling

Technicians were identified using purposive sampling [37]. An initial list of technicians operating in the area of Toamasina was used to recruit potential participants based on our previous work there (M. Usowicz, personal communication, 28 June 2019) [34]. Using a snowball sampling, each participant was also asked to refer other technicians, or refer the research team to local hardware stores where technicians obtain materials. There is no available estimate for the technician population in Toamasina; therefore, the sample size needed could not be determined and recruitment was guided by the concept of theoretical saturation. A sample size of 10–15 is usually sufficient to achieve theoretical saturation for most marketing domains (e.g., perceived benefits and barriers, responsiveness to a proposed behavior change) [38,39].

### 2.4. Recruitment

Participants were recruited with the help of a local non-governmental organization. A recruitment questionnaire was administered in person and responses recorded on paper and entered in Qualtrics [40], an online survey and analysis tool, upon return to the office. Recruitment continued until the research team deemed that no new technician names were emerging from the interviews.

### 2.5. Data Collection

Eligible technicians were asked to participate in a one-time, in-depth interview. A semi-structured interview guide was used to conduct the interviews. The guide was developed to gain an in-depth understanding of the reasons behind Pb use or non-use by technicians in the region. The interviews covered the following domains: training received; installation/repair work practices; detail on types of materials used, preferences, and availability; techniques of installation and repairs; knowledge of water safety and water quality; channels through which they receive training or information; knowledge of the health effects of Pb; and barriers to and facilitators of the use of Pb-free components. The guide was pretested with two technicians and revised prior to continuing recruitment. Interviews were recorded, transcribed verbatim in Malagasy, and translated into English. A sample (*n* = 2) of transcribed and translated transcripts were back-translated (i.e., Malagasy to English and back to Malagasy) to ensure accuracy of translation.

### 2.6. Data Analysis

English-language transcripts were coded using MaxQDA 2020 [41] for qualitative applied thematic analysis. A codebook that included a priori and emerging codes was developed by the research team. Transcripts were coded by three members of the study team using MaxQDA software. To ensure that codes had been applied consistently by all three coders, intercoder reliability was assessed by calculating Cohen’s kappa coefficient, which is read on a scale of −1 to 1 where 0.61–0.80 is substantial and 0.81–1 is almost perfect agreement [42]. The kappa coefficient was found to be >0.80 for all pairs of coders. Thematic analysis was conducted on coded transcripts to identify key patterns. A “doer/non-doer” lens was adopted during data analysis, meaning that responses from technicians who still use Pb in repairs and new pump installations (non-doers) were compared to responses for technicians who only use Pb-free components (doers) to determine underlying differences between the two groups [35,36].

## 3. Results

An initial recruitment survey identified 49 technicians operating in Toamasina. Of these, 25 indicated having received formal training on the risks associated with Pb in water from the USF and were therefore excluded from the study as per the eligibility criteria. Of the remaining technicians, 18 were available to participate in an in-depth individual interview.

### 3.1. Sample Description

All 18 technicians self-identified as male. Mean age was 34 years. On average, technicians had 12 years of experience working in the field. Additional information on the sample is reported in Table 1. The study sample was based within 11 fokontany/neighborhoods and two arrondissements/administrative districts, Ankirihiry and Tanambao V. Technicians were generally trained informally by transferring skills from another profession and learning from family and friends. When asked about what they enjoy about their work, participants focused on their role in the community and the pride in providing water for other people who rely on their expertise.

### 3.2. Current Behavioral Uptake

Of the 18 technicians interviewed, six reported not using Pb anymore (doer) in their repair and installation practice. Twelve participants reported still using Pb (non-doer). The breakdown of these two groups is presented in the following sections and in the summary table (Table 2).

### 3.3. Reported Factors Influencing Behavioral Uptake

Doer analysis. Participants who reported not using Pb, meaning those already doing the behavior of using lead-free components, mentioned either the excessive cost (*n* = 3), lack of availability (*n* = 2), and/or knowledge of its negative health effects (*n* = 2) as main reasons for the switch to lead-free components. Participants who mentioned the consequences on individual well-being as a reason for not using Pb shared that receiving information on Pb’s adverse health effects was sufficient to prompt behavior change.

Non-doer analysis. Participants who still used Pb shared that several of its characteristics make it particularly suitable for certain components of the pump. One key aspect was the greater density of this metal, which was associated with creating a better seal. As shared by some technicians:

“… And so usually, when you use leaded weights for the valves, the system seals better and keeps water because the lead is heavy. Because, once you pump, the valve opens up, and the water would flow, and when you stop pumping, the valve would seal and keeps some water above it; but if you use iron, since iron is lighter in weight, the valve would not seal properly.” (male technician, age 28)

Along with the material’s density, technicians reported that Pb is easy to melt and mold to create pump components. As shared by one technician:

“[Iron is more complicated to work with] you [need] to buy bolts to keep the iron weight in place; but with lead, you just need to melt it, meaning you melt it then afterward you put it directly on the valve. That’s the difference between the two elements.” (male technician, age 28)

Use of Pb is also engrained in their repair and installation practices, as this is how they have been taught to make pumps. One participant called the use of Pb “the Malagasy way” of making pitcher pumps (male technician, age 26).

“Because the older people used it, that’s why we use it too. It makes the pump last for such a long time, as I said that older people used it.” (male technician, age 47)

Compared with other metals, such as iron and steel, technicians said that Pb does not rust and is simpler to use. Technicians reported that the challenges in finding Pb in recent years and its high cost sometimes drove them to switch to an alternative metal. Iron seemed to be the preferred metal when they cannot find Pb, as six out of the 11 technicians reported using it.

“No! Lead is the main one, then iron sometimes! Lead is the one we usually use, but we use iron when we can’t find lead.” (male technician, age 28)

### 3.4. Perceived Benefits and Barriers of Using Lead-Free Components

Doers. Technicians who had already made the switch to lead-free components mentioned the savings on the overall cost of making or repairing a pump, and the ease of finding components as main benefits. Three out of six doers also reported the health benefits of going Pb-free, but only one of them mentioned these effects as the sole advantage of the switch.

When asked about the barriers that other technicians may encounter in adopting the new behavior, doers said that some technicians do not know how to make repairs without using Pb and are not particularly keen to change their methods as Pb is easier for them to use. Lack of knowledge of the health effects of Pb was also mentioned, with one participant saying that some technicians may know about the issues around Pb but may not believe it has an impact on the health of individuals.

Non-doers. When asked what they would see as beneficial in switching to lead-free components, technicians who were still using Pb said that they believed it would improve health outcomes. One technician specifically mentioned children as a main beneficiary of this potential change. Only one technician mentioned that it would be beneficial to reduce the cost of manufacturing the pump, and that it would be easier to find components given that Pb is becoming increasingly less available.

With respect to barriers of behavior change, most non-doers mentioned those similar to those of the doers (e.g., Pb is easier to use, they are not aware of any health concerns). The main difference between doers and non-doers is that some technicians in the latter group believed it is not possible to create a pump that is fully lead-free, as some components cannot be created with other metals, and that going lead-free would affect the long-term quality and lifespan of the pump.

### 3.5. Perceived Motivators for Change

Doers. Technicians suggested focusing on the following potential motivators to encourage their peers to switch to lead-free components: showing technicians how easy it is to find and use iron (*n* = 2) and explaining the adverse health effects (*n* = 4).

Non-doers. Similarly, non-doers believe that technicians like themselves may be motivated to change if they knew their work helps protect the health of the public by protecting their clients against the dangerous effects of Pb (*n* = 5), and if they were shown how to correctly make the switch to lead-free components (*n* = 4).

### 3.6. Knowledge of Water Quality and Safety

Technicians were asked about the quality of water in Toamasina and over half (*n* = 11) expressed concern. This concern was overshadowed by the relief of having access to, and the availability of, water as it is needed for a wide variety of health and economic benefits. When asked about the quality of the water provided by the pumps, technicians shared that they evaluate the water quality based on the appearance (*n* = 12), smell (*n* = 9), and taste (*n* = 7). It should be noted that for a pitcher pump, the practical pumping limit is approximately 9 m [43]. This limit, along with the high-water table and high density of latrines in the area, can lead to further contamination of the water being drawn for use from chemical and microbial contaminates [44]. Technicians were knowledgeable of areas with water quality issues, such as the neighborhood of Andranomadio, which was mentioned by six out of the 18. There is an understanding that water quality suffers in the area; however, technicians feel that it is not their responsibility or that they do not have the proper information to convey to the public. As one technician stated:

“I can’t guarantee the quality of water. But I just make the pump.” (male technician, age 31)

### 3.7. Information-Seeking Behaviors and Preferences for Communication Channels

Technicians did not seem to have a reliable and established source of information regarding water quality and safety issues. Several participants (*n* = 6) explicitly mentioned relying on their own expertise and abilities to guarantee a quality product for their clients. Peers and colleagues were the most mentioned source of information on these matters (*n* = 7). Participants shared that spokespersons, who are peers and colleagues, should accompany their message with practical demonstrations on how to create and use components that are lead-free.

### 3.8. Preferences for Interventions

**Figure 3:**
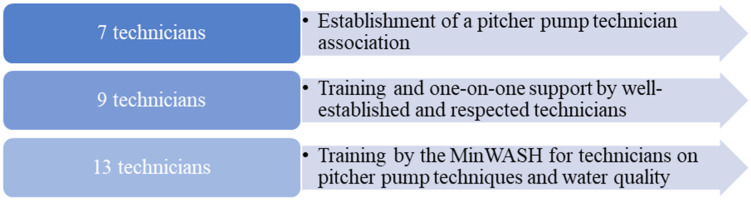
Three types of interventions were reviewed by technicians during semi-structured in-depth interviews with respect to encouraging the switch to non-lead components. The number of participants represents those seeing the benefit in the given intervention.

Technicians also provided feedback on which interventions they would see as beneficial in promoting a behavior shift among their peers (Figure 3). The most popular proposal was the development of a training curriculum lead by the Ministries of Water and Health to assist them with knowledge and practical applications to improve water quality. Technicians were also responsive to the idea of one-on-one support and training from more experienced peers, and to the establishment of an association as a focal point for the development of initiatives and idea-sharing.

## 4. Discussion

This study aimed to understand the mechanisms guiding usage of Pb parts for pitcher pumps in Toamasina, Madagascar, and to explore technicians’ willingness to switch to non-leaded components that would lead to an improved product. The community-based, user-centric approach adopted in this study using social marketing describes the level of knowledge, barriers, and facilitators of behavior change related to pump manufacturing. Additionally, the midstream approach adopted by this study intends to effectively address the problem at a social ecology level, ultimately leading to reduced Pb exposure for local households.

Overall, technicians did not exhibit sophisticated knowledge of the specific consequences of Pb contamination on individual health. Technicians who had already switched to lead-free components had a general notion that Pb is dangerous for health, but not clear knowledge of the adverse effects. Even without an in-depth understanding of the mechanisms of the health effects of Pb, raising awareness through targeted education and information was considered enough by many technicians to prompt behavior change among their peers: non-doers felt compelled to change their practice after being told about the health consequences of Pb exposure. This finding is quite interesting in that social marketing and other behavior change approaches espouse that knowledge does not lead to behavior change, and that people act irrationally and not in line with common sense [45,46]. However, in this instance it seems that there is a threshold that is reached early that makes the decision to change based on health-related information the easier choice [47].

Additional barriers to switching to lead-free components were identified. These included a lack of technical expertise on how to build certain components of the pump without Pb and, in a few cases, clients’ requests to use Pb. Technicians already using lead-free components shared that their choice to switch was facilitated by the fact that they knew of other materials that worked well to build pump components and other technicians that have made the switch to lead-free components. This was coupled with the fact that Pb is often hard to find and expensive. This finding suggests that besides improving awareness of health issues, a successful intervention should provide technicians with appropriate techniques to switch to lead-free components (as well as options for materials achieving similar results to Pb in terms of molding/melting). Such a change would require a shift in supply of materials or a change in policy that further reduces the availability of Pb in the market (or the prices of Pb components) in a way that reduces its competitive advantage compared with other materials such as iron or steel.

Findings around barriers were mirrored by the benefits technicians identified in lead-free pump production. The doers focused on the cost savings and wider availability of lead-free components, while the non-doers pointed to the health benefits of switching to other metals. This could speak to the temporality of the information about Pb. While information on health benefits of lead-free pumps may be sufficient to prompt change at the beginning, technicians who do not use Pb perceive that their choice is beneficial also from an economic and convenience perspective. This makes the behavior change sustainable. Therefore, to produce long-term change, technicians should also receive training on techniques that can help them go lead-free and information on the economic advantage of this choice. In this sense, future studies should look at behavior change in this area as a dynamic process [48,49]. 

Besides informing the type of information and skill needs that should be met, findings from this formative research show how to best convey that information to the midstream audience of pump technicians. Technicians seemed to favor exchange of information among peers when it comes to specific techniques to work on pitcher pumps. However, they believed the Ministries of Water and Health are well positioned to provide information on water quality more broadly. Though these channels were identified, technicians were generally skeptical of information they receive from anyone. This insight points us towards developing a strategy that reaches out to “market mavens” [50]. These are highly connected, well-respected technicians, who serve as disseminators of information, have firsthand experience of making the switch, and are in a position to influence other technicians.

The findings identified in this study will inform the development of a social marketing campaign and behavior change intervention in Madagascar. The main insight from the research findings indicates that technicians wish to be respected in the community, and to be considered a professional and an innovator. As such, the direction for the campaign will follow the approach of providing the expert-voice from respected technicians on how to make the switch, and how lead-free is the best practice in the future. This follows the nudging tactics of herding, the creation of social norms, and using messengers that are respected by the audience [51,52]. Additionally, the campaign will awaken the curiosity of the technicians by sharing salient facts about the dangers of Pb and how it is an outdated practice. The campaign will be rolled out in phases by local organizations and experienced technicians. The initial focus will be on branding and awareness (information), a second phase on providing skills and tools on making the switch to lead-free (education), and the final phase will focus on dissemination to make the intervention sustainable (practicability).

This study shows how a midstream approach to social marketing can uncover factors that drive behaviors around use of Pb in the installation and repair of pitcher pumps. Any intervention aiming to improve water quality through removal of Pb should consider the barriers and benefits that service-level personnel face when making decisions around the manufacturing of components and the requisite purchase of metals and incorporate those considerations into product and program development. This paper provides insights into the trade-off between the benefits of going lead-free and the barriers to making that switch as experienced by pitcher pump technicians in Toamasina, Madagascar. This midstream approach to social marketing can be applied to informal sectors around the world where Pb has been identified (e.g., cooking vessels in Cameroon [53] and spices in Bangladesh [54]) and where supplemental water systems are prevalent in order to help meet targets of the Sustainable Development Goals (SDGs) related to provision of safe water and good health and well-being.

## 5. Conclusions

This study illustrates the importance of investigating the mechanisms of behavior change and understanding what factors encourage the adoption of a new behavior, product, or service. When a new product or service is developed and tested for efficacy, an important element is to investigate the human element and see how the user will react to the product and adapt or not adapt it in their routine. As illustrated in this paper, engineering WASH projects can be complemented with approaches that center on the experience of the user. Social marketing is a documented strategy for understanding behavior and developing interventions that are multifaceted and responsive to the user. Additionally, this paper makes an important contribution to social marketing and engineering by moving the discussion to midstream approaches that focus on service personnel, community organizations, and other related groups in a position to influence. These groups often serve as intermediaries between the system and the end-user and determine and support behavior change.

## Figures and Tables

**Table 1:** Socio-demographic and knowledge variable breakdown of surveyed technician sample.

Socio-Demographic and Knowledge Variable	Percentage (%) of Participants
**Primary language**	
Malagasy	71%
French	29%
**Status**	
Married or in a domestic partnership	82%
Single	18%
**Household size**	
1–2 people	18%
3–4 people	47%
5–6 people	18%
7+ people	18%
Previously heard information about water quality issues and water contamination	65%
**Training received**	
Apprenticeship	6%
Transferred skills from another profession (i.e., welding)	82%
Formal technician training	6%
More than one source of training	6%
Presence of another technician in the family	65%
Average number of new pumps installed each month	3
Average number of pumps repaired each month	3.5

**Table 2:** Doer/Non-doer analysis.

	Doers	Non-Doers
	Technicians Who Are Not Using Pb	Technicians Who Use Pb Components
Reported motivators for their current behavioral choice	Participants mentioned: excessive cost of Pb (*n* = 4) lack of availability of Pb (*n* = 2) desire to avoid negative health effects (*n* = 6)	Participants mentioned: physical properties of Pb (e.g., density, malleability, and it does not rust) (*n* = 7)
Benefits of lead-free components	Participants mentioned: cost savings from using cheaper metals (*n* = 4) wider availability/lack of supply issues with other metals (*n* = 2) health benefits (*n* = 2)	Participants mentioned: health benefits (*n* = 7) cost savings (*n* = 1)
Barriers to lead-free components	Participants mentioned: lack of knowledge of how to use other metals and lack of willingness to learn (*n* = 4) lack of knowledge of health effects of Pb (*n* = 2) metals such as iron are more difficult to use for manufacturing of pump components (*n* = 1)	Participants mentioned: lack of knowledge of how to use other metals (*n* = 1) metals such as iron are more difficult to use for manufacturing of pump components (*n* = 4) concern that the quality of pump manufacturing will worsen with a switch to non-lead (*n* = 1) lack of awareness of the adverse effects of Pb (*n* = 2)
Perceived motivators for change to lead-free components	Participants perceived that technicians would feel motivated to change: if they were told they can easily use iron (*n* = 1) if they were made aware of the adverse effects of Pb on health (*n* = 4) if they were told that other metals are easier to find (*n* = 1)	Participants perceived that technicians would feel motivated to change: if they were told they would protect public health (*n* = 6) if they were shown how to easily switch to other metals (*n* = 3)

## Data Availability

The redacted data presented in this study are available on request from the corresponding author. The data are not publicly available due to privacy restrictions.
